# Genome-wide association study identifies SNPs in the MHC class II loci that are associated with self-reported history of whooping cough

**DOI:** 10.1093/hmg/ddv293

**Published:** 2015-07-30

**Authors:** George McMahon, Susan M. Ring, George Davey-Smith, Nicholas J. Timpson

**Affiliations:** 1 School of Social and Community Medicine and; 2 MRC Integrative Epidemiology Unit, University of Bristol, Bristol, UK

## Abstract

Whooping cough is currently seeing resurgence in countries despite high vaccine coverage. There is considerable variation in subject-specific response to infection and vaccine efficacy, but little is known about the role of human genetics. We carried out a case–control genome-wide association study of adult or parent-reported history of whooping cough in two cohorts from the UK: the ALSPAC cohort and the 1958 British Birth Cohort (815/758 cases and 6341/4308 controls, respectively). We also imputed HLA alleles using dense SNP data in the MHC region and carried out gene-based and gene-set tests of association and estimated the amount of additive genetic variation explained by common SNPs. We observed a novel association at SNPs in the MHC class II region in both cohorts [lead SNP rs9271768 after meta-analysis, odds ratio [95% confidence intervals (CIs)] 1.47 (1.35, 1.6), *P*-value 1.21E − 18]. Multiple strong associations were also observed at alleles at the HLA class II loci. The majority of these associations were explained by the lead SNP rs9271768. Gene-based and gene-set tests and estimates of explainable common genetic variation could not establish the presence of additional associations in our sample. Genetic variation at the MHC class II region plays a role in susceptibility to whooping cough. These findings provide additional perspective on mechanisms of whooping cough infection and vaccine efficacy.

## Introduction

Whooping cough, also known as pertussis, is a highly contagious infection of the respiratory tract. It is transmitted from person to person by coughing or sneezing and caused primarily by the gram-negative bacteria *Bordetella pertussis.* Without intervention it is a highly morbid infection in infants, and in the pre-vaccine era, it was responsible for 10 000 deaths per year in the UK (1). Where vaccination is not routine, whooping cough is still a major source of mortality leading to 195 000 deaths in 2008 (2).

In developed countries, the incidence of whooping cough has been effectively controlled by vaccination. However, it is endemic with periodic epidemics every 3–4 years. Improving vaccination efficacy represents a sustained goal to improve the control of whooping cough (3,4) and thereby reducing associated mortality, morbidity and economic costs (5,6). Current vaccination does not provide complete protection (7) and immunity wanes with time. Furthermore, there is a requirement to combine multiple strategies to improve coverage among the most vulnerable, including an accelerated schedule and preschool booster (8), cocooning (9) and immunization of expectant mothers (10). There is also growing evidence of a recent increasing numbers of cases, particularly in infants and among adolescents and adults (8,11,12). Among the latter group this increase may be partly due to better surveillance and reporting (11,13) but among very young infants notifications are already well established and less likely to explain an increase (14). These factors have led to re-evaluation of the effectiveness of current vaccination efforts (15,16).

Studies suggest a genetic component exists for susceptibility to whooping cough. A twin study in The Gambia indicated interferon-gamma and interleukin-13 responses to *B. pertussis* antigens are heritable (17). Murine models of pertussis infection show that mice with a functional mutation in the *TLR4* gene are more susceptible to infection (18,19) and a candidate gene approach has indicated that variation in the human *TLR-4* gene influences antibody response to pertussis vaccine (20–22).

Although these studies are promising they have been limited in replicating initial associations by small sample sizes. For other infectious diseases, the genome-wide association study (GWAS) has been successful in discovering and replicating several genetic associations (23,24). A difficulty in examining a role for common genetic variants in causing whooping cough is the accurate identification of a large enough numbers of cases to robustly identify associations of small to moderate effect expected from common variant analysis (25). This leads to the dilemma of using measures of whooping cough collected in large prospective cohorts, which may rely on historic data or retrospectively self-reported history of whooping cough from a time when whooping cough was more common. Self-reported measures, in particular, can suffer from recall bias on the exact nature of the outcome introducing unwanted heterogeneity into the analysis leading to a loss of power and difficulties in the interpretation of the nature of an observational association.

Despite these potential difficulties, genome-wide analysis is a potentially powerful approach to identifying associations between risk whooping cough and common genetic variation, and this approach has yet to be tested in this context. Therefore, this study aimed to carry out a GWAS using data from two independent cohorts from the UK (1573 cases and 10 649 controls combined) to attempt to identify associations at individual loci and to determine the degree to which common genetic variation influences reported history of whooping cough.

## Results

Overall the proportion of participants who reported a history of whooping cough was 0.11 (95% CIs 0.10–0.12) in ALSPAC and 0.15 (95% CIs 0.14–0.16) in the 1958 birth cohort (Table 1). In ALSPAC, all participants were female and 72% were born between 1958 and 1968 whereas in the 1958 birth cohort, 50.6% were female and all were born in 1958. In ALSPAC, the proportion of participants who reported a positive history varied across year of birth, being highest for participants born pre-1958 and after 1968 and lowest between 1958 and 1967. In the 1958 birth cohort, history of whooping cough was more common in girls than boys. In ALSPAC, there was good agreement between self-reported history of whooping cough reported 8 years after initial data collection. 81 and 89% of subjects with repeat data on positive or negative self-reported history of whooping cough reported consistently at both time periods (Table 2, Cohen's kappa 0.56).

**Table 1.** History of whooping cough by sex, year of birth and region of birth

	ALSPAC				1958 Birth Cohort			
	Overall	Cases	Controls	*P*-value	Overall	Cases	Controls	*P*-value
Sex								
Male	—	—	—	—	0.49 (2499)	0.14 (342)	0.86 (2157)	0.013
Female	1 (7156)	0.11 (815)	0.89 (6341)		0.51 (2567)	0.16 (416)	0.84 (2151)	
Total	1 (7156)	0.11 (815)	0.89 (6341)		1 (5066)	0.15 (758)	0.85 (4308)	
Year of birth								
1948–1952	0.02 (112)	0.28 (31)	0.72 (81)	<0.001		—	—	—
1953–1957	0.11 (773)	0.18 (138)	0.82 (635)			—	—	
1958–1962	0.32 (2303)	0.11 (250)	0.89 (2053)		1 (5066)	0.15 (758)	0.85 (4308)	
1963–1967	0.4 (2831)	0.09 (258)	0.91 (2573)			—	—	
1968–1972	0.14 (977)	0.11 (112)	0.89 (865)			—	—	
1973–1976	0.02 (160)	0.16 (26)	0.84 (134)			—	—	
Total	1 (7156)	0.11 (815)	0.89 (6341)		1 (5066)	0.15 (758)	0.85 (4308)	

The proportions (counts) of participants within those designated as ‘cases’ and ‘controls’ and overall are shown within strata with a *P*-value for a chi-square test of association where possible. The symbol ‘—’ indicates no data. A u-shaped association with year of birth was confirmed by second-order polynomial regression (*P* = 2E − 10 for addition of the squared term).

**Table 2.** Agreement between self-reported history of whooping cough in the ALSPAC cohort

Time		+ 8 years			Total
	Response	Yes	No	Unsure	
0	Yes	444 (0.81)	75	28	547
	No	138	3608 (0.89)	330	4076
	Total	582	3683	358	4623

64% **(**4623/7156) subjects included in the main analysis had a repeat measure available. Proportions indicate subjects with consistent measures across time points. Similar levels of agreement were observed using all subjects with repeat measures (*n* = 7084).

**Figure 1.**
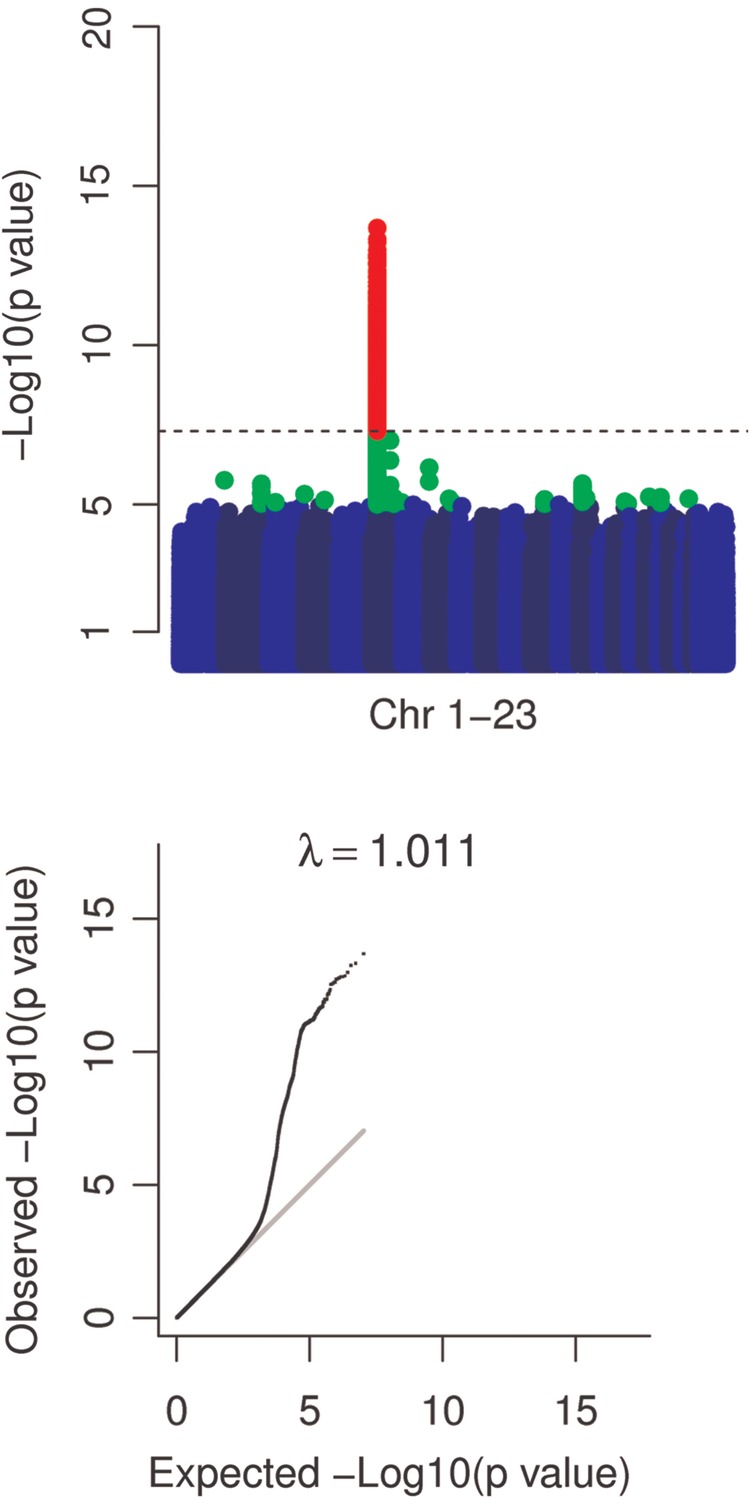
Manhattan and QQ plots. Manhattan plots with markers *P* < 1.E − 5 in green and *P* < 5.E − 8 in red indicate a strong signal on chromosome 6. QQ plots of test statistics show inflation of associations at lower *P*-values. A grey line indicates a straight line relationship. The genomic inflation factor (*λ*) is shown in text above the QQ plot. Removal of the extended MHC region resulted in a genomic inflation factor of 1.007. A dashed line indicates *P* = 5.E − 8.

**Figure 2.**
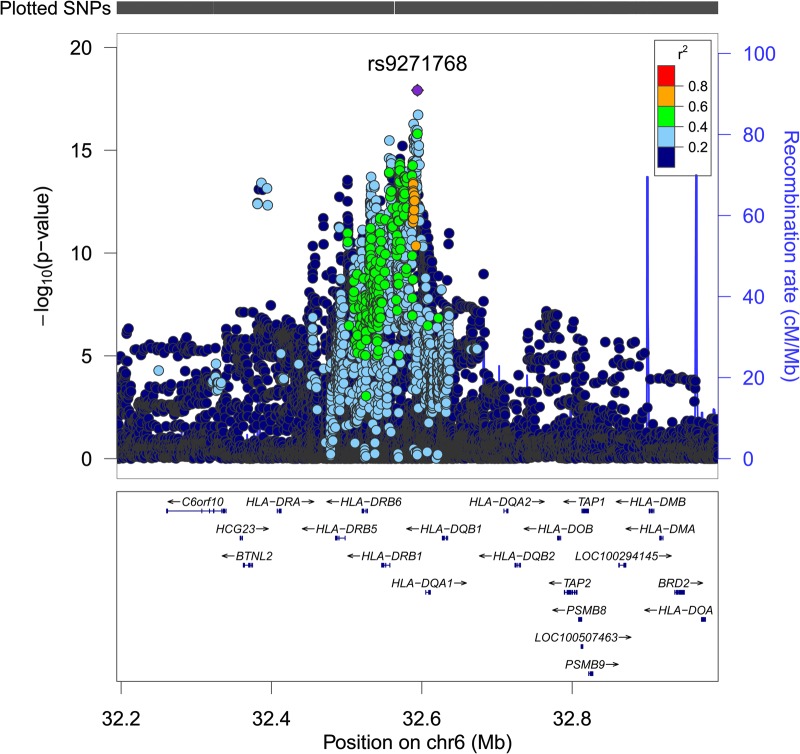
Regional plot of lead association signal. *P*-values (on a −log10 scale) after meta-analysis are shown over the MHC class II region. Each SNP is coloured according to the degree of linkage disequilibrium with the lead SNP rs9271768 (shown as a purple diamond). Linkage disequilibrium values are from the 1000 Genomes (March 2012 release) European population.

We carried out a genome-wide association analysis in the ALSPAC cohort and observed a single strong association peak on chromosome 6, with a complementary excess at the extremes of the test statistic distribution and an absence of systematic genome-wide inflation (Fig. 1). The lead SNP, located in the MHC class II region, was rs2760994 [odds ratio (95% CIs) 1.54 (1.37, 1.72), *P*-value 2.1E − 14]. A strong association signal was also observed using subjects with repeated measures collected 8 years later [odds ratio (95% CIs) 1.38 (1.21,1.57), *P*-value 1.2E − 06, *n* = 4,265]. We followed this up by conducting a genome-wide association analysis in the 1958 birth cohort and observed a complementary association signal [odds ratio (95% CIs) 1.27 (1.13, 1.44), *P*-value 9.6E − 05, Supplementary Material, Figs S1–S3]. After meta-analysis, the lead SNP was located 20 KB away at rs9271768 [effect allele G, odds ratio (95% CIs) 1.47 (1.35, 1.6), *P*-value 1.21E − 18] in an intergenic region between *HLA-DRB1* and *HLA-DQA1* (Table 3 and Fig. 2).

**Table 3.** Lead SNP associations

			ALSPAC				1958 birth cohort				Meta-analysis			
SNP	Position (build 37)	Effect/non-effect allele	Effect allele frequency	Imputation quality	OR (95% CIs)	*P*-value	Effect allele frequency	Imputation quality	OR (95% CIs)	*P*-value	Direction	OR (95% CIs)	*P*-value	Q *P*-value
rs2760994	3 257 4308	T/C	0.60	0.98	1.54 (1.37, 1.72)	2.11E − 14	0.58	0.87	1.27 (1.13, 1.44)	9.60E − 05	++	1.41 (1.3, 1.53)	6.11E − 16	0.0242
rs9271768	3 259 4188	G/A	0.52	0.88	1.54 (1.37, 1.72)	1.05E − 13	0.57	0.78	1.39 (1.23, 1.59)	3.23E − 07	++	1.47 (1.35, 1.6)	1.21E − 18	0.272

Odds ratios (OR) and confidence intervals (CIs) are shown for a one unit increase in the number of effect alleles at each SNP within each cohort and after meta-analysis.

We split participants in each data set into groups based on region of birth and re-examined an association between SNP rs9271768 and risk of history of whooping cough because alleles at the HLA region exhibit geographical variation (26), which could correlate with risk of exposure to pertussis (Table 4). While in ALSPAC there was evidence of a weak association between history of whooping cough and region of birth (*P* = 0.06), no strong evidence of stratification by rs9271768 genotype was found (*P* = 0.175) and adjustment for region of birth had little or no effect on the strength of the original principal component-corrected association between rs9271768 and history of whooping cough (*P* = 1.4E − 13 after adjustment). Similarly, in the 1958 birth cohort, a moderate association between history of whooping cough and region of birth (*P* = 0.001) was observed, no further evidence of stratification by rs9271768 genotype was found (*P* = 0.1) and adjustment for region of birth reduced only marginally the original strength of principal component-corrected association between rs9271768 and history of whooping cough (*P* = 4.30E − 06).

**Table 4.** Test of confounding of lead association by region of birth

Study	Region of birth	History of whooping cough				rs9271768 genotype					rs9271768 G counts (positive history of whooping cough/total)			Regression coefficient^a^	*P*-value^b^	Overall *P*-value^c^
		Overall^d^	cases	controls	*P*-value^e^	AA	AG	GG	Total	*P*-value^f^	0	1	2			
ALSPAC	East/West Ridings	0.02 (155)	0.11 (17)	0.89 (138)	0.06	0.258 (40)	0.477 (74)	0.265 (41)	155 (1)	0.1751	0.075 (3/40)	0.081 (6/74)	0.195 (8/41)	0.06	0.083	1.40E − 13
	Eastern	0.04 (280)	0.1 (27)	0.9 (253)		0.246 (69)	0.496 (139)	0.257 (72)	280 (1)		0.072 (5/69)	0.086 (12/139)	0.139 (10/72)	0.033	0.18	
	London	0.06 (403)	0.12 (50)	0.88 (353)		0.213 (86)	0.514 (207)	0.273 (110)	403 (1)		0.081 (7/86)	0.082 (17/207)	0.236 (26/110)	0.082	5.01E − 04	
	Midlands	0.04 (270)	0.14 (37)	0.86 (233)		0.207 (56)	0.496 (134)	0.296 (80)	270 (1)		0.125 (7/56)	0.104 (14/134)	0.2 (16/80)	0.043	0.152	
	North Midland	0.01 (68)	0.06 (4)	0.94 (64)		0.235 (16)	0.515 (35)	0.25 (17)	68 (1)		0.188 (3/16)	0 (0/35)	0.059 (1/17)	-0.062	0.13	
	Northern	0.02 (120)	0.19 (23)	0.81 (97)		0.292 (35)	0.525 (63)	0.183 (22)	120 (1)		0.143 (5/35)	0.143 (9/63)	0.409 (9/22)	0.117	0.028	
	Northwest	0.04 (234)	0.12 (29)	0.88 (205)		0.231 (54)	0.526 (123)	0.244 (57)	234 (1)		0.056 (3/54)	0.13 (16/123)	0.175 (10/57)	0.06	0.057	
	Scotland	0.02 (103)	0.17 (18)	0.83 (85)		0.282 (29)	0.476 (49)	0.243 (25)	103 (1)		0.069 (2/29)	0.184 (9/49)	0.28 (7/25)	0.106	0.042	
	Southeast	0.01 (93)	0.11 (10)	0.89 (83)		0.183 (17)	0.441 (41)	0.376 (35)	93 (1)		0.118 (2/17)	0.073 (3/41)	0.143 (5/35)	0.022	0.624	
	Southern	0.04 (244)	0.09 (21)	0.91 (223)		0.213 (52)	0.561 (137)	0.225 (55)	244 (1)		0.038 (2/52)	0.088 (12/137)	0.127 (7/55)	0.044	0.103	
	Southwest	0.66 (4193)	0.11 (458)	0.89 (3735)		0.22 (923)	0.505 (2118)	0.275 (1152)	4193 (1)		0.07 (65/923)	0.107 (227/2118)	0.144 (166/1152)	0.037	8.17E − 08	
	Wales	0.03 (205)	0.1 (21)	0.9 (184)		0.195 (40)	0.454 (93)	0.351 (72)	205 (1)		0.1 (4/40)	0.097 (9/93)	0.111 (8/72)	0.007	0.818	
	Total	1 (6368)	0.11 (715)	0.89 (5653)		0.223 (1417)	0.505 (3213)	0.273 (1738)	6368 (1)		0.076 (108/1417)	0.104 (334/3213)	0.157 (273/1738)	0.041	3.09E − 13	
1958 birth cohort	East/West Ridings	0.09 (461)	0.15 (70)	0.85 (391)	0.001	0.152 (70)	0.551 (254)	0.297 (137)	461 (1)	0.1	0.1(7/70)	0.13(33/254)	0.219(30/137)	0.065	0.011	4.30E − 06
	Eastern	0.08 (381)	0.13 (49)	0.87 (332)		0.136 (52)	0.483 (184)	0.381 (145)	381 (1)		0.115(6/52)	0.12(22/184)	0.145(21/145)	0.017	0.492	
	London	0.12 (592)	0.11 (68)	0.89 (524)		0.142 (84)	0.5 (296)	0.358 (212)	592 (1)		0.06(5/84)	0.108(32/296)	0.146(31/212)	0.042	0.031	
	Midlands	0.1 (503)	0.17 (88)	0.83 (415)		0.151 (76)	0.499 (251)	0.35 (176)	503 (1)		0.132(10/76)	0.175(44/251)	0.193(34/176)	0.028	0.262	
	North Midland	0.08 (407)	0.13 (51)	0.87 (356)		0.14 (57)	0.496 (202)	0.364 (148)	407 (1)		0.053(3/57)	0.139(28/202)	0.135(20/148)	0.03	0.214	
	Northern	0.08 (420)	0.14 (59)	0.86 (361)		0.167 (70)	0.464 (195)	0.369 (155)	420 (1)		0.1(7/70)	0.128(25/195)	0.174(27/155)	0.039	0.108	
	Northwest	0.12 (609)	0.16 (96)	0.84 (513)		0.186 (113)	0.478 (291)	0.337 (205)	609 (1)		0.133(15/113)	0.141(41/291)	0.195(40/205)	0.035	0.099	
	Scotland	0.11 (545)	0.21 (114)	0.79 (431)		0.172 (94)	0.523 (285)	0.305 (166)	545 (1)		0.17(16/94)	0.218(62/285)	0.217(36/166)	0.02	0.443	
	Southeast	0.05 (251)	0.14 (34)	0.86 (217)		0.127 (32)	0.454 (114)	0.418 (105)	251 (1)		0.062(2/32)	0.105(12/114)	0.19(20/105)	0.07	0.028	
	Southern	0.06 (299)	0.14 (42)	0.86 (257)		0.127 (38)	0.488 (146)	0.385 (115)	299 (1)		0.053(2/38)	0.144(21/146)	0.165(19/115)	0.046	0.124	
	Southwest	0.06 (295)	0.12 (35)	0.88 (260)		0.136 (40)	0.508 (150)	0.356 (105)	295 (1)		0.125(5/40)	0.113(17/150)	0.124(13/105)	0.002	0.938	
	Wales	0.05 (248)	0.16 (40)	0.84 (208)		0.169 (42)	0.472 (117)	0.359 (89)	248 (1)		0.119(5/42)	0.179(21/117)	0.157(14/89)	0.012	0.727	
	Total	1 (5011)	0.15 (746)	0.85 (4265)		0.153 (768)	0.496 (2485)	0.351 (1758)	5011 (1)		0.108 (83/768)	0.144 (358/2485)	0.173 (305/1758)	0.032	1.41E − 05	

Three tests of association were carried out: between region of birth and history of whooping cough, region of birth and rs9271768 genotype and between rs9271768 genotype and history of whooping cough stratified by region of birth. The distribution of proportions and their corresponding counts are shown within cells.

^a^Rate of change of proportion with number of G alleles of rs9271768.

^b^Test of trend between counts of the G allele of rs9271768 and history of whooping cough.

^c^Overall test of trend between counts of the G allele of rs9271768 and history of whooping cough after adjusting for region of birth.

^d^Proportion of participants in each strata.

^e^Chi-square test of the association between region of birth and counts of cases and controls.

^f^Chi-square test of the association between region of birth and counts of rs9271768 genotype.

We also conditioned on SNP rs9271768 in both cohorts and meta-analysed the results for SNPs within the xMHC but did not obtain clear evidence of further association (*P* > 1.E − 6 after meta-analysis, Supplementary Material, Table S3). The lead SNPs within each cohort while located within 20 KB of each other differed slightly in their location and were in weak-to-moderate linkage disequilibrium (Supplementary Material, Table S4). Therefore, we examined whether they represented distinct signals. Conditional analysis indicated that correcting for the lead SNP in the ALSPAC cohort could account for any additional signal at the lead 1958 BC cohort SNP and vice versa (Supplementary Material, Table S5). We also examined epistasis among the lead SNPs. The inclusion of two-way interaction terms did not result in a better fitting model than a model including two SNPs alone (*P*-value >0.2). At these SNPs, conditional analysis in the ALSPAC cohort, stratifying on year of birth as an instrument for vaccination status to test the hypothesis of environmental mediation by this factor, did not indicate evidence of effect modification (Supplementary Material, Table S6).

Classical HLA alleles imputed to four-digit resolution using either HLA-IMP*02 or SNP2HLA in ALSPAC showed concordant effects sizes (Supplementary Material, Fig. S4). Haplotype analysis of imputed HLA alleles resulted in a strong association peak over the HLA class II alleles in both cohorts (Supplementary Material, Tables S7 and S8) with evidence of association at HLA-DQA, HLA-DQB, HLA-DRB1 and HLA-DRB3/4/5. The most strongly associated locus was HLA-DQB1 (*P*-value 7.8E − 16) with strongest individual effects for alleles HLA-DQB1*0602, HLA-DQB1*0501 and HLA-DRB1*0301 (*P*-value <1.E − 5). Addition of the HLA class II alleles to a model already including the lead SNP rs9271768 indicated the majority of signal at these alleles could be attributed to the lead SNP association (Supplementary Material, Table S7). Strong association signals for amino acids within the HLA class II alleles were also observed. The strongest evidence of association was for a set of amino acids at position 13 in HLA-DRB1 (presence of either serine or histidine versus all others, *P*-value 7.86.E − 17). Conditioning on lead SNP rs9271768 resulted in most of the signal being explained (*P*-value 1.03E − 03, Supplementary Material, Table S9).

Estimates of the variance explained of all SNPs within the extended MHC were 0.007 (SE 0.003, *P*-value 9.E − 5) in the ALSPAC cohort and 0.007 (SE 0.004, *P*-value 0.01) in the 1958 birth cohort, respectively, whereas the variability explained by all SNPs genome-wide were 0.11 (SE 0.05, *P*-value 0.01) and 0 (S.E. 0.07, *P*-value 0.5). Genome-wide gene-based tests of association confirmed the single SNP analysis giving a strong association signal at the MHC class II loci *HLA-DQB1, HLA-DRB1, HLA-DQA1* and *TAP2* whereas outside the MHC region, gene-based and gene-set analysis did not result in identifiable additional associations (Supplementary Material, Tables S10 and S11). Candidate SNP analysis at SNPs at the *TLR4* gene (rs2770150, rs4986790), which have been previously shown to be associated with antibody response to pertussis toxin, did not show evidence of association with reported history of whooping cough in this sample (Supplementary Material, Table S12).

## Discussion

This study applied genome-wide association analysis to reported history of whooping cough. We observed evidence of association within the MHC class II region with the strongest signal at SNP rs9271768. The genomic location of the lead association signal suggests a role for antigen binding and presentation in its aetiology as extracellular proteins from invading pathogenic bacteria are primarily processed via class II molecules. Biochemical data indicate molecules encoded by the HLA class II loci respond biologically to pertussis. Cell surface expression of HLA-D molecules on human monocytes is modulated by pertussis infection (27), and *B. pertussis* peptides require *HLA-DR1* to elicit a response (28). Furthermore, genetic variation at *HLA-DQ1* has been shown to modify CD4^+^ T-cell response, upon stimulation with pertactin *in vitro* (29).

We observed strong overlapping signals in both cohorts where lead SNPs within each cohort and after meta-analysis were located within 20 KB of each other in the MHC class II region. Pinpointing the causal mutation definitively may have been made more challenging owing to the complex patterns of linkage disequilibrium across the MHC. Imputed alleles at the MHC class II loci were found to be strongly associated with our outcome although no more strongly associated than the lead SNP. Identifying the nature of the genetic effect in the region and its potential relationship with HLA antigen loading represents an important next step in understanding this association.

Our attempts to confirm whether additional variation was present in our sample by testing all SNPs simultaneously and using genome-wide gene-based tests and gene-set tests were inconclusive. This sample may have been underpowered to detect additional associations. There are alternative explanations. Historical accounts indicate that pertussis emerged in Europe only in the last 500 years (30), and sequence diversity measures indicate that *B. pertussis* has a relatively recent evolutionary origin (31). A possible explanation for the lack of additional genetic associations might be that too little time has passed for *B. pertussis* to exert an appreciable selective pressure. Another possible explanation is that host adaptation by *B. pertussis* is thought to have been mediated by large-scale gene loss and inactivation (32), which may have led to a small number of pathogenic loci that are responsible for determining host susceptibility. Either way the estimates of variance explained by all SNPs represent a lower bound for the total additive genetic variability explained owing to incomplete LD between directly typed SNPs and causative mutations (33), and by examining all common SNPs jointly, power to detect the influence of a small number of moderate to weak effects is less compared with the joint effect of true causal loci alone. Furthermore, our results are not well powered to inform on non-additive and/or rare variations. Nevertheless, our results suggest that effect sizes at the MHC class II region are relatively larger than those from other genomic regions in these population-based samples.

Genome-wide association studies can be biased by population stratification and errors in phenotype definition (34). We applied two methods of correction for potential bias owing to population substructure; principal component analysis of genome-wide data, a standard approach in GWAS that adjusts cases and controls for differences in ancestry genetic variation (35) and traditional epidemiological tests of confounding. Both methods indicated a similar strength of association between SNP rs9271768 and self-reported history of whooping cough, which indicates population stratification is unlikely to have led to a spurious association in these samples. We have been careful to describe our phenotype as self-reported history of whooping cough to acknowledge its potential heterogeneity. Obtaining a large enough number of cases to robustly determine a common genetic association was complicated by the widespread and effective control of pertussis in developed countries. In practice, this meant we were limited to cohorts with historical data. Furthermore, data were collected by either self-retrospective report or parent reported questionnaire. While a proportion of these would have been doctor-diagnosed cases, some cases of whooping cough involve *Bordetella parapertussis* (36–38) and some viruses such as adenovirus and parainfluenza virus can cause pertussis-like syndrome in children leading to differential diagnosis (39,40).

The above limitations may mean that, while we believe this is a replicable association, robust to confounding, in theory it is possible the association is partly driven by a whooping cough like syndrome caused by agents different to *B. pertussis* or possibly the observed association acts mechanistically through vaccine-driven susceptibility (by effect modification).

In summary, our data indicate that common genetic variation in the MHC class II region influences susceptibility to self-reported history of whooping cough. Examination of this genetic variant in laboratory-confirmed cases and its possible correlation with pertussis endophenotypes or response to vaccination is an important next step to understand this association.

## Material and Methods

### Study cohorts

#### ALSPAC

The Avon Longitudinal Study of Children and Parents (ALSPAC) is a longitudinal birth cohort with a sampling frame of all pregnant women with an expected delivery date between April 1991 and December 1992 within a well-defined geographic area in the Southwest UK including a major city (Bristol). Initially, 14 541 pregnant women (and their children) were recruited and over the 20 years of follow-up, the majority are still participating in questionnaires that are sent out at regular intervals and by attending study clinics (41). The study website contains details of all the data that are available through a fully searchable data dictionary (http://www.bris.ac.uk/alspac/researchers/data-access/data-dictionary/). Mothers completed a self-report questionnaire at approximately the twelfth week of pregnancy, which included a question on history of whooping cough infection. Mothers were asked ‘Have you ever had any of the following infections’ and were presented with a list of common infections including whooping cough to which they could respond either ‘Yes’, ‘No never’ or ‘I don't know’. The same question was asked again in a separate questionnaire 8 years later. Participants’ region of birth was identified from the question ‘Where were your parents living at the time you were born?’ with spaces left blank for town, county and country. Postal area was extracted from this information and placed in categories matching the 1958 birth cohort definitions. Ethical approval for the study was obtained from the ALSPAC Ethics and Law Committee and the Local Research Ethics Committees.

#### 1958 birth cohort

The 1958 British Birth cohort (1958 birth cohort) also known as the National Child Development Study is a longitudinal birth cohort with a sampling frame of all those born in a particular week in March in 1958 in England, Scotland and Wales. Initially, 17 000 people were recruited, the majority of whom have participated in regular follow-up surveys and a biomedical survey (42). Detailed documentation about the 1958 Birth Cohort, including a fully searchable data dictionary, can be found online (http://www.cls.ioe.ac.uk/). When participants were aged between 7 and 8 years, their parent completed a questionnaire by interview, which included the question ‘What infectious diseases has the child had, and at what ages’. Parents were asked to select from a list of common childhood diseases including whooping cough and could indicate ‘Yes’, ‘No’ and ‘Don't know’. Region of residence of Great Britain was defined by postcode (26).

### Genotype quality control and imputation

PLINK (43) (v1.07) was used to carry out quality control filtering on directly typed data from both cohorts. SNPs were removed if they had >5% missingness or Hardy–Weinberg equilibrium *P* < 1.E − 6. Additionally, SNPs with MAF of <1% were removed. Samples were excluded if they had >5% missingness, indeterminate X-chromosome heterozygosity or extreme autosomal heterozygosity. Analysis was restricted to individuals with European ancestry. Samples showing evidence of population stratification were identified by multidimensional scaling of genome-wide identity-by-state pairwise distances using the CEU, YRI, JPT and CHB HapMap populations as a reference and were then excluded. Cryptic relatedness was defined as a Pi hat value of >0.125, which is expected to correspond to roughly 12.5% of alleles being shared IBD or relatedness at a first-cousin level. Phasing was carried out in Shapeit2 (44) (v2.r644), and data were imputed to the 1000 Genomes Phase I Integrated Release (version 3) in IMPUTE2(v2.3) (45).

#### ALSPAC

Genome-wide data of common genetic variation were generated at The Centre National de Génotypage (CNG) on the Illumina Human660W-Quad array, and genotypes were called with Illumina GenomeStudio. Out of an initial set of 10 015 subjects and 557 124 directly genotyped SNPs, 9048 subjects and 526 688 SNPs passed the quality control filters (including related subjects that passed all other quality control thresholds). We combined 477 482 SNPs of these subjects with genotype data of a sample of 9115 children to allow the use of a large number of duos by the phasing algorithm to increase quality of phased haplotypes and downstream imputation. This results in the removal SNPs genotypes with a poor concordance across data sets, which could be completely captured by increasing the missingness filter 5 to 1% (an additional 11 396 SNPs). We also removed a further 321 subjects owing to potential id mismatches when assessed using duo data. This resulted in a data set of 17 842 subjects containing 6305 duos and 465 740 SNPs (112 were removed during liftover and 234 were out of HWE after combination). This gave 8196 eligible mothers with available genotype data after exclusion of related subjects. A total of 7156 unrelated subjects had both phenotype and genotype data.

#### 1958 birth cohort

Genome-wide data of common genetic variation were prepared by combining genotype data from three studies that have previously genotyped subjects from the 1958 Birth Cohort; the Wellcome Trust Case Control Consortium (WTCCC), the GABRIEL Consortium (CNG) and the Type 1 Diabetes Genetic Consortium (T1DGC). The details of the genotype data used in this study and their quality control are given in Supplementary Material, Section S1. A total of 5847 samples and 504,606 SNPs were available after quality control filters were applied, and 5066 subjects had both phenotype and genotype data.

### HLA imputation

We imputed the MHC class I loci *HLA-A*, *HLA-B*, *HLA-C* and class II loci *HLA-DQA1*, *HLA-DQB1*, *HLA-DPB1*, *HLA-DRB1*, *HLA-DRB3, HLA-DRB4, HLA-DRB5* using HLA*IMP:02 (46) accessed 10 June 2013. After quality control, 1908 (1958 Birth Cohort) and 2061 (ALSPAC) directly genotyped SNPs in the extended MHC (xMHC), chromosome 6 from base pair position (build 36) 25 921 129 to 33 535 328 (46) were included. Imputation resulted in 158 and 155 HLA alleles from the ALSPAC and 1958 Birth Cohort data, respectively. Alleles were imputed to a four-digit level, apart from HLA-DRB1 paralogs, which were to a two-digit level. We removed genotypes with a posterior probability under 0.70. We re-imputed the both data sets with SNP2HLA (47) using the default settings, accessed 04 June 2015 to confirm our first imputation. This resulted in 64 four-digit HLA alleles all of which were imputed with HLA*IMP:02. We also re-analysed both of our data sets using data generated in SNP2HLA because of the broad set of additional variations in the region it can impute: specifically, markers for the presence/absence of an amino acid residue, markers for the presence/absence of groups of amino acids residues given a multi-allelic amino acid position, HLA intragenic SNPs and insertion/deletions.

### Statistical analysis

A genome-wide association analysis was carried out in each set of directly genotyped and imputed bi-allelic markers within each cohort using an additive logistic model, log(odds) = *a* + *BX* + *c*, where *X* is the allelic dosage of the effect allele (0, 1 or 2 copies of *b*) at the *n*th SNP and *c* are study-specific covariates. These were year of birth (first and second order) and the top ten principal components of population ancestry (PCs) in ALSPAC and sex, a study-specific indicator and the ten PCs in the 1958 Birth Cohort. Within-cohort association analyses were carried out in SNPTEST (v2.4.0) (48). Effects were matched by marker name and were combined using inverse variance-based meta-analysis in METAL (49). Regional plots were drawn in LocusZoom (50). *P*-values interactions between lead SNPs after association analysis or between lead SNPs and year of birth were obtained from the difference in deviances between models including main effects versus main effects plus their interactions.

A potential limitation to the analysis of questionnaire-derived data concerning infection is the distinction between exposure and susceptibility. Given the focus on susceptibility, here we undertook sensitivity analyses with the aim to remove residual structure from our data, which may be correlated with differential infection exposure. We examined the association at the lead SNP after meta-analysis within region of birth strata using a test of trend of proportions similar to the Cochran–Armitage test of trend apart from the factor *N*/(*N* − 1), which is appropriate when *N* is small in particular when data are subdivided into strata and computed a test of overall trend across strata (51,52).

#### Analysis of imputed HLA alleles

In haplotype analysis to examine whether associations with SNP data could be mapped to variation in the classical HLA alleles, we used multiple logistical regression that would allow the addition of covariates and assumed additivity in the effects at a haplotype at a locus (53). We used the same within-cohort covariates as described earlier. Statistical details of this analysis are given in Supplementary Material, Section S5. Additional variation in the region was analysed using imputed allele dosage in PLINK and a logistic regression model for the presence/absence of a marker plus the same within-cohort covariates as described earlier.

#### GCTA, gene-based and gene-set analysis

We set out to assess whether additional regions of the genome were associated with our phenotype, which could only be observed by assessing SNPs jointly. The variance explained by all directly genotyped SNPs and SNPs within the extended MHC region correcting for the same covariates as single SNP analysis was estimated using GCTA (54). Gene associations were calculated in a Versatile Gene-Based Test for Genome-wide Association Studies (VEGAS, v0.8.27) (55). An empirical *P*-value of *P* < 2.8–6 was taken as a conservative threshold for identifying sites of interest (a Bonferroni-corrected threshold of 0.05/17 787 tested genes). Gene-set analysis, to identify sets of variants affecting susceptibility across genes, was carried out in Meta-Analysis Gene-set Enrichment of variaNT Associations (MAGENTA, v2.4) using all available gene sets (56). A Bonferroni correction was used to correct for multiple testing owing to the number of gene sets being analysed (*P* < 1.55E − 5, *n* = 3216). SNP *P*-values after meta-analysis were used for both gene-based and gene-set analyses. For gene-based and pathway analysis, we filtered out SNPs with an INFO score of <0.5 and MAF of <0.01.

## Supplementary Material

Supplementary Material is available at *HMG* online.

## Funding

The UK Medical Research Council and the Wellcome Trust (Grant ref: 102215/2/13/2) and the University of Bristol provide core support for ALSPAC. G.D.S., N.J.T. and S.M.R. work in the Medical Research Council Integrative Epidemiology Unit at the University of Bristol, which is supported by the Medical Research Council (grant numbers MC_UU_12013/1 and MC_UU_12013/3) and the University of Bristol. This work made use of data and samples generated by the 1958 Birth Cohort (NCDS). Access to these resources was enabled via the 58READIE Project funded by Wellcome Trust and Medical Research Council (grant numbers WT095219MA and G1001799). A full list of the financial, institutional and personal contributions to the development of the 1958 Birth Cohort Biomedical resource is available at http://www2.le.ac.uk/projects/birthcohort. Genotyping was undertaken as part of the Wellcome Trust Case-Control Consortium (WTCCC) under Wellcome Trust award 076113, and a full list of the investigators who contributed to the generation of the data is available at www.wtccc.org.uk. Funding to pay the Open Access publication charges for this article was provided by the Wellcome Trust.

## Supplementary Material

Supplementary Data
