# Supplementary material for: Genome-wide association study identifies SNPs in the MHC class II loci that are associated with self-reported history of whooping cough
Source: Hum Mol Genet. 2015 Jul 30;24(20):5930–9. doi: 10.1093/hmg/ddv293 (PMC4581602; doi:10.1093/hmg/ddv293)
Supplement: Supplementary Data [file supp_24_20_5930__index.html]

Genome-wide association study identifies SNPs in the MHC class II loci that are associated with self-reported history of whooping cough — Genome-wide association study identifies SNPs in the MHC class II loci that are associated with self-reported history of whooping cough — Genome-wide association study identifies SNPs in the MHC class II loci that are associated with self-reported history of whooping cough — Supplementary Data 

# Genome-wide association study identifies SNPs in the MHC class II loci that are associated with self-reported history of whooping cough

## Supplementary Data

Supplementary Data

- Supplementary Data - Docx file
- Supplementary Tables - xlsx file
