# Supplementary material for: Genome-wide association study identifies SNPs in the MHC class II loci that are associated with self-reported history of whooping cough
Source: Hum Mol Genet. 2015 Jul 30;24(20):5930–9. doi: 10.1093/hmg/ddv293 (PMC4581602; doi:10.1093/hmg/ddv293)
Supplement: Supplementary Data [file supp_ddv293_ddv293supp.docx]

Genome-wide association study identifies SNPs in the MHC class II loci that are associated with self-reported history of whooping cough.

George Mc Mahon^1^, Susan M Ring^1,2^, George Davey-Smith^1,2^, Nicholas J Timpson^1,2^*.

^1^School of Social and Community Medicine, University of Bristol, Bristol, UK

^2^MRC Integrative Epidemiology Unit, University of Bristol, Bristol, UK

Section S1: Merging of three datasets from the 1958 birth cohort genotype data

**Table S1** Quality control of 1958 birth cohort genome-wide SNP data

Section S2: Examination of population stratification

**Figure S1** Manhattan and QQ plots of GWAS summary statistics

Section S3: SNP association results

**Figure S2** Regional plot of MHC class II region ALSPAC data

**Figure S3** Regional plot of MHC class II region 1958 birth cohort data

**Table S2** Summary statistics for SNPs P < 1.e-5 after meta-analysis

Section S4: Conditional SNP association results

**Table S3** Association and meta-analysis results of SNP in the xMHC conditional on SNP rs9271768

**Table S4** Lead SNPs within cohort

**Table S5** Conditional analysis of within cohort lead SNPs

**Table S6** Associations between lead SNPs and year of birth as an instrument for vaccination status in the ALSPAC cohort

Section S5: Analysis of imputed HLA allele data

**Figure S4** Comparison of imputed HLA effects using SNP2HLA

**Table S7** Per loci effects (4-digit alleles)

**Table S8** Per allele effects (4-digit alleles)

**Table S9** Per allele effects (additional variation in the MHC)

Section S6: Gene-based and gene-set analysis

**Table S10** Gene based tests

**Table S11** Gene set enrichment analysis

Section S7: Further association analysis

**Table S12** Assessment of loci previously associated with whooping cough endophenotypes

Merging of three datasets from the 1958 birth cohort genotype data

Standard quality control measures described in the main text were applied to each 1958 birth cohort dataset separately resulting in 510,328 overlapping SNPs and 6,023 subjects. Data combined from different data sets could lead to chip specific genotyping error that is too low to be identified by the conventional quality control steps and may correlate with disease status (e.g. if one dataset genotyped more cases) leading to inflation of type I error. We scanned for evidence of this by carrying out an association analysis of each dataset against the other two at overlapping SNPs. Genomic control indicated acceptable levels inflation (0.995 – 1.01 for all three comparisons). As expected, there were a minority (N = 38) instances of SNPs with allele frequencies that differed between datasets. We observed a relationship between SNP missingness and chip specific differences, where 32 out of 38 SNPs displayed over 2% missingness. Missingness as a possible source of allele frequency differences between subjects genotyped on different chips has been previously reported([1](#_ENREF_1)). SNPs with a missingness of > 2% in any dataset were removed (N = 5313) and since the remaining number of SNPs with a P < 1.E-5 were small (N = 14) they were excluded and the datasets were merged. Since we used an arbitrary threshold (P < 1.E-5) to determine if allele frequencies differed between 1958 birth cohort datasets, we also examined whether chip status correlated with differences between SNP genotypes cumulatively in GCTA. The variance explained by all directly genotyped SNPs for each study within the 1958 birth cohort data compared to the other two was then estimated. In each analysis estimates overlapped zero ; variance exlained (SE) WTCCC vs. CNG 0.065 (0.3), WTCCC vs. T1DGC 0 (0.3) and CNG vs. T1DGC 0 (0.5). After examining the effect of chip status on SNP genotype, individual dataset level quality control measures were re-applied after the data was merged and results in a minor number of further exclusions (Table S1).

**Table S1** **Quality control of 1958 birth cohort genome-wide SNP data.** Quality control was applied in two stages. In stage1, quality control was applied to each dataset separately while in stage 2 further quality control was applied after merging the individual datasets that involved comparing allele frequencies across samples. Stage 1 quality control was reapplied after stage 2.

|  |  | **Stage 1: QC of individual datasets** | | | | **Stage 2: QC of combined dataset** | | | | | | | | |
| --- | --- | --- | --- | --- | --- | --- | --- | --- | --- | --- | --- | --- | --- | --- |
| **Dataset** | **Genotyping chip** | **N samples before qc** | **N samples after qc** | **N SNPs before qc** | **N SNPs after qc** | **Additional SNP missingness filter** | **Case control SNPs (P < 1.E-5)** | **Cryptic relatedess across datasets** | **ID mismatches** | **Application of individual dataset qc level measures** | **N samples before qc** | **N samples after qc** | **N SNPs before qc** | **N SNPs after qc** |
| WTCCC | Illumina 1.2M | 2919 | 2740 | 1157986 | 948222 | 0.02  (N = 5,313) | N = 14 | 11 (relatedness > 0.1) | N = 6 | 159 samples and 388 SNPs | 6023 | 5,847 | 510,328 | 504,606 |
| CNG | Illumina 610-Quad | 872 | 817 | 582892 | 546477 |  |  |  |  |  |  |  |  |  |
| T1DG | Infinium HumanHap 550K v3 | 2592 | 2472 | 561303 | 533859 |  |  |  |  |  |  |  |  |  |

Examination of population stratification

After association analysis we were interested to examine if there was inflation among SNP test statistics and therefore calculated the genomic inflation factor (lambda, as $\frac{{median(\chi}_{1})}{0.455}$) and drew QQ plots. To help visualise P values from association analysis, Manhattan plots were also drawn. These graphs are shown in Figure S1 for the 1958 birth cohort and after meta-analysis (the corresponding graphs using ALSPAC data are shown in Figure 1). No evidence of excess of false positives was observed; the genomic inflation factors were reasonable (1.005-1.011), the QQ plots showed prominent departures from the null in the extreme tails of the distribution but no early separation of the null and observed test statistics and Manhattan plots showed one single spike.

**Figure S1 Manhattan and QQ plots of GWAS summary statistics 1958 birth cohort and meta-analysis data.** Top panel: Manhattan plots with markers P < 1.E-5 in green and P < 5.E-8 in red indicate a single peak on chromosome 6. Bottom panel: QQ plots of test statistics show inflation of associations at lower P values. A grey line indicates a straight line relationship. The genomic inflation factor (λ), shown in text above each plot indicates little genome-wide inflation of test statistics. A dashed line indicates P = 5.E-8.


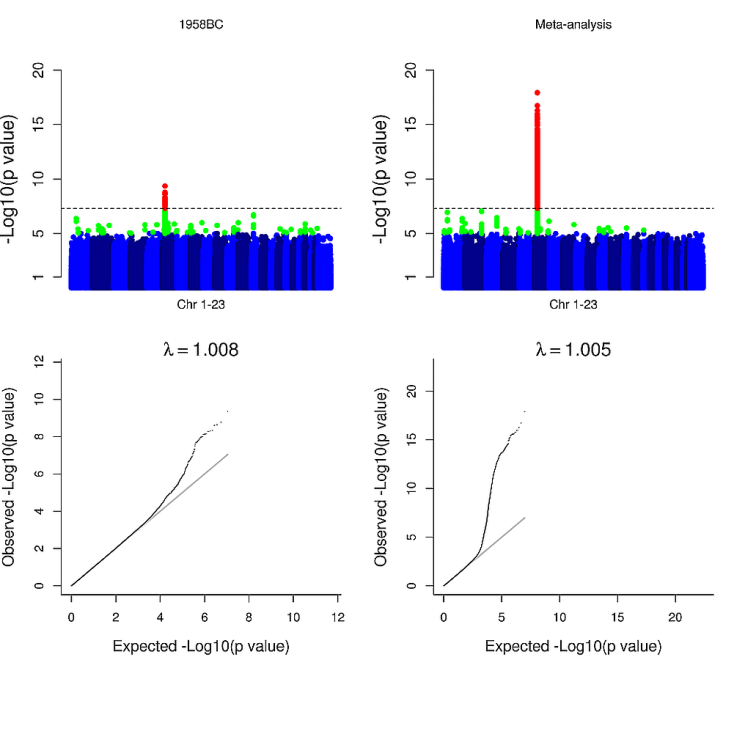


SNP association results

**Figure S2 Regional plot of MHC class II region ALSPAC data.** P values (on a -log10 scale) are shown plotted against base pair position. Each SNP is coloured according to the degree of linkage disequilibrium with the lead SNP (shown as a purple diamond). Linkage disequilibrium values are from the 1000 Genomes (March 2012 release) European population.

**

**

**Figure S3 Regional plot of MHC class II region 1958 birth cohort data.** P values (on a -log10 scale) are shown plotted against base pair position. Each SNP is coloured according to the degree of linkage disequilibrium with the lead SNP (shown as a purple diamond). Linkage disequilibrium values are from the 1000 Genomes (March 2012 release) European population.





**Table S2** **Summary statistics for SNPs P < 1.e-5 after meta-analysis** (see separate file).

Conditional SNP association results

**Table S3 Association and meta-analysis results of SNP in the xMHC conditional on SNP rs9271768** (see separate file)

**Table S4 Within cohort lead SNP associations.** Summary statistics for the lead SNP within each cohort and their linkage disequilibrium (squared correlation based on genotypic allele counts, R^2^) and intergenic distance in kilobases (KB). Corresponding lower and upper triangles indicate linkage disequilibrium in ALSPAC and the 1958 BC respectively.

|  |  |  | **ALSPAC summary stats** | | | | **1958 BC summary stats** | | | | **R^2^ (Distance in KB)** | | |
| --- | --- | --- | --- | --- | --- | --- | --- | --- | --- | --- | --- | --- | --- |
|  | **Top hit** | **Effect/**  **non-effect allele** | **OR (95% CIs)** | **P value** | **Effect allele frequency** | **Imputation quality** | **OR (95% CIs)** | **P value** | **Effect allele frequency** | **Imputation quality** | **rs2760994** | **rs111696694** | **rs9271768** |
| **ALSPAC** | **rs2760994** | T/C | 1.54 (1.37, 1.72) | 2.11E-14 | 0.60 | 0.99 | 1.27 (1.13, 1.44) | 9.60E-05 | 0.58 | 0.87 | 1 | 0.31 (14.81) | 0.35 (19.88) |
| **1958 BC** | **rs111696694** | C/A | 1.37 (1.23, 1.53) | 1.92E-08 | 0.35 | 0.98 | 1.45 (1.29, 1.62) | 4.51E-10 | 0.34 | 0.94 | 0.35 (14.81) | 1 | 0.25 (5.07) |
| **Meta-analysis** | **rs9271768** | G/A | 1.54 (1.37, 1.72) | 1.05E-13 | 0.52 | 0.88 | 1.39 (1.23, 1.59) | 3.23E-07 | 0.57 | 0.78 | 0.65 (19.88) | 0.28 (5.07) | 1 |

**Table S5 Conditional analysis of within cohort lead SNPs.** Summary statistics for a model that includes both lead SNPs for each sample.

|  | **SNPs** | **Effect/**  **non-effect allele** | **OR (95% CIs)** | **P value** |
| --- | --- | --- | --- | --- |
| **ALSPAC** | **rs2760994** | T/C | 1.46 (1.36, 1.56) | 3.56E-08 |
|  | **rs111696694** | C/A | 1.08 (1.01, 1.15) | 2.56E-01 |
| **1958 BC** | **rs2760994** | T/C | 1.02 (0.95, 1.09) | 7.77E-01 |
|  | **rs111696694** | C/A | 1.4 (1.31, 1.5) | 1.34E-06 |

**Table S6 Associations between lead SNPs and year of birth as an instrument for vaccination status in the ALSPAC cohort.** Association analysis at SNPs resulted in overlapping odds ratios across strata. Inclusion of two-way interactions terms between year of birth and SNP genotype or (year of birth)^2^ and SNP genotype were non-significant (P > 0.2, data not shown).

| **ALSPAC** |  | **Year of Birth (as instrument for vaccination)** | | | |
| --- | --- | --- | --- | --- | --- |
|  |  | **Pre 1957 (n = 618)** | | **Post 1957 (n = 6538)** | |
|  | **SNP** | **OR (95% CIs)** | **P value** | **OR (95% CIs)** | **P value** |
|  | **rs2760994** | 1.73 (1.28,2.35) | 3.8E-04 | 1.5 (1.33,1.69) | 4.58E-11 |
|  | **rs111696694** | 1.56 (1.17,2.09) | 2.4E-03 | 1.32 (1.18,1.48) | 2.7E-06 |
|  | **rs9271768** | 1.54 (1.15,2.06) | 3.6E-03 | 1.48 (1.32,1.66) | 3.33E-11 |

Analysis of imputed HLA allele data

**Figure S4 Comparison of imputed HLA effects using SNP2HLA.** Effect sizes (beta/SE) for the 64 four digits classical HLA alleles generated using HLA*IMP:02 or SNP2HLA. Tests of association were undertaken for the presence of absence of each allele.


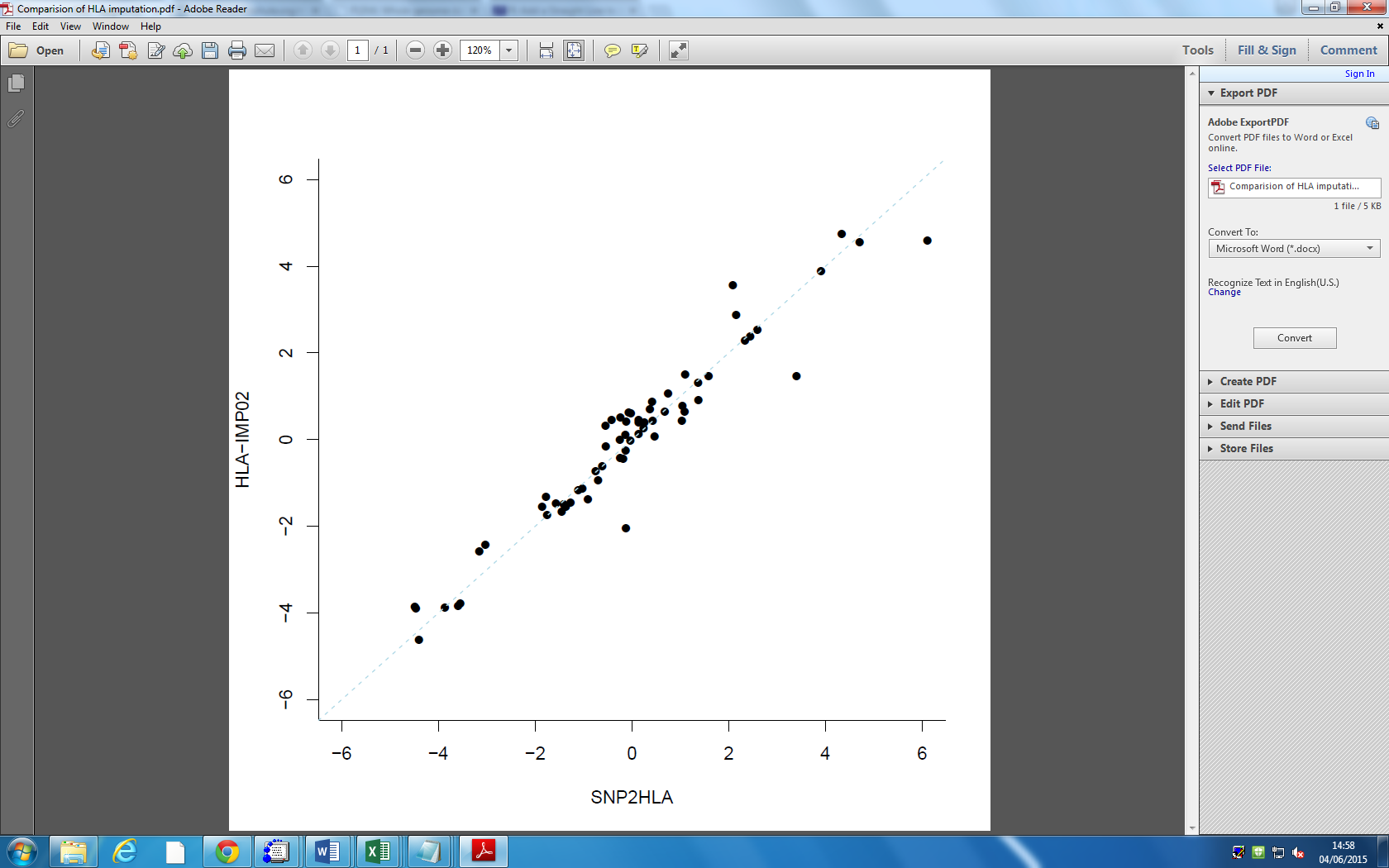


To analyse the multi-allelic HLA loci, we used a logistic regression model that would allow the addition of covariates and assumed additively in the effects at a particular haplotype. The model was as follows, log(odds) = a + ΣB_k_Z_k_ + C + e where Z_k_ is an indicator variable of the number of copies of the kth haplotype at a locus where the most frequent allele in the ALSPAC cohort has been arbitrarily set as the reference allele. B_k_ represents the increase in log(odds) for one copy of the kth haplotype and one copy of the reference allele compared to two copies of the reference allele. C indicates study specific covariates listed for the genome-wide association analysis. Individual effects were combined by fixed effects meta-analysis. An overall test of association for a particular locus was obtained by a likelihood ratio test between the above model including m - 1 indicator variables for each m haplotypes versus the model of study specific covariates which is distributed as a chi square variable with m – 1 degrees of freedom (df)([2](#_ENREF_2)). P values from the chi-square tests were combined across cohorts using Fisher’s method([3](#_ENREF_3)).

**Table S7 Per loci effects (4-digit alleles).** Overall tests of association at each imputed locus are shown with a chi-square value and degree of freedom (df) and P value of a likelihood ratio test for inclusion of four digit alleles (or two digit alleles at the HLA-DRB1 paralogs).

|  |  | **ALSPAC** | | | **1958 birth cohort** | | | **Meta-analysis** | |
| --- | --- | --- | --- | --- | --- | --- | --- | --- | --- |
|  | **Locus** | **Chi sq** | **df** | **P value** | **Chi sq** | **df** | **P value** | **Chi sq** | **P value** |
| **Unconditional** | **A** | 99.67 | 18 | 3.7E-01 | 45.60 | 18 | 3.2E-01 | 4.31 | 3.7E-01 |
|  | **B** | 118.03 | 36 | 9.0E-02 | 60.40 | 34 | 5.0E-01 | 6.20 | 1.8E-01 |
|  | **C** | 108.58 | 18 | 5.4E-02 | 46.89 | 18 | 5.9E-01 | 6.89 | 1.4E-01 |
|  | **DPB1** | 58.12 | 6 | 5.0E-01 | 26.25 | 6 | 5.5E-01 | 2.61 | 6.2E-01 |
|  | **DQA1** | 122.42 | 7 | 1.6E-09 | 55.80 | 7 | 1.2E-02 | 49.36 | 4.9E-10 |
|  | **DQB1** | 159.76 | 13 | 1.1E-12 | 74.46 | 13 | 2.0E-05 | 76.83 | 7.8E-16 |
|  | **DRB1** | 123.20 | 22 | 1.2E-07 | 63.11 | 21 | 3.8E-03 | 42.92 | 1.1E-08 |
|  | **DRB 3/4/5** | 114.00 | 7 | 1.2E-07 | 71.02 | 6 | 5.5E-06 | 56.10 | 1.9E-11 |
| **Conditional on rs9271768** | **A** | 143.57 | 18 | 8.0E-01 | 60.45 | 18 | 4.0E-01 | 2.26 | 6.9E-01 |
|  | **B** | 156.70 | 36 | 7.2E-01 | 71.77 | 34 | 6.2E-01 | 1.62 | 8.0E-01 |
|  | **C** | 144.30 | 18 | 6.9E-01 | 69.24 | 18 | 4.3E-01 | 2.41 | 6.6E-01 |
|  | **DPB1** | 89.17 | 6 | 6.5E-01 | 45.71 | 6 | 4.5E-01 | 2.45 | 6.5E-01 |
|  | **DQA1** | 122.11 | 7 | 6.2E-02 | 61.95 | 7 | 1.9E-01 | 8.93 | 6.3E-02 |
|  | **DQB1** | 159.76 | 13 | 1.4E-03 | 75.26 | 13 | 2.7E-02 | 20.31 | 4.3E-04 |
|  | **DRB1** | 122.78 | 22 | 5.2E-02 | 63.92 | 21 | 4.8E-02 | 12.00 | 1.7E-02 |
|  | **DRB 3/4/5** | 120.73 | 7 | 3.6E-01 | 71.39 | 6 | 4.2E-04 | 17.62 | 1.5E-03 |

**Table S8** **Per allele effects (4-digit alleles).** Individual effect sizes for the classical HLA alleles at four digit resolution for each allele are shown as the log odds ratio against the reference allele (BETA) and their standard errors (SE) and P value for each cohort and after meta-analysis (see separate file).

**Table S9** **Per allele effects (additional variation in the MHC).** Individual effect sizes for the additional sets of variation in the MHC region specifically; markers for the presence/absence of an amino acid residue, markers for the presence/absence of groups of amino acids residues, HLA intragenic SNPs, insertion/deletions and classical HLA alleles at two digit resolution. Each allele was also conditioned on the lead SNP within each cohort and after meta-analysis. Alleles denoted by P/A indicate presence/absence of the allele/amino acid (see separate file).

Gene-based and gene-set analysis.

**Table S10 Gene based tests outside the MHC**. Genes with an empirical P value of < 1.E-3 after analysis in VEGAS are shown with data on their chromosomal location, gene name and lowest scoring SNP +/- 50 kb of their boundaries. Outside the MHC region no gene based test of association reached significance (P < 2.8E-6). Gene sets (P < 0.05 in gene set analysis) and their respective databases are aslo listed. nSNPs, number of tested SNPs; P value, gene based P value; Best-SNP, lowest score SNP at locus; SNP-P value, P value for lowest scoring SNP.

| Chr | Gene | nSNPs | Start | Stop | Pvalue | Best-SNP | SNP-pvalue | Pathway |
| --- | --- | --- | --- | --- | --- | --- | --- | --- |
| 1 | C8A | 179 | 57093066 | 57156482 | 9.E-05 | rs737189 | 4.81E-05 | complement activation (GO term), complement activation, classical pathway (GO term), cytolysis (GO term), |
| 1 | CEP170 | 92 | 241354352 | 241485331 | 9.E-04 | rs10926958 | 2.65E-04 |  |
| 1 | OR2M3 | 113 | 246432992 | 246433931 | 8.E-04 | rs11204613 | 1.00E-04 | g-protein coupled receptor protein signaling pathway (GO term), |
| 1 | OR2M4 | 97 | 246468853 | 246469789 | 3.E-04 | rs11204613 | 1.00E-04 | g-protein coupled receptor protein signaling pathway (GO term), |
| 2 | ZC3H6 | 67 | 112749648 | 112814111 | 5.E-04 | rs4525706 | 1.53E-04 |  |
| 3 | ACAD9 | 112 | 130081022 | 130114647 | 8.E-04 | rs11926639 | 8.80E-07 | electron transport (PANTHER_BIOLOGICAL_PROCESS), |
| 3 | KIAA1257 | 62 | 130172471 | 130195676 | 1.E-04 | rs11916893 | 5.09E-07 |  |
| 3 | CCDC48 | 42 | 130231981 | 130242273 | 2.E-04 | rs11916893 | 5.09E-07 |  |
| 7 | VPS37D | 58 | 72720109 | 72724376 | 5.E-04 | rs12531606 | 1.61E-04 |  |
| 7 | DNAJC30 | 57 | 72733183 | 72735717 | 5.E-04 | rs12531606 | 1.61E-04 |  |
| 7 | WBSCR22 | 63 | 72735833 | 72750478 | 5.E-04 | rs12531606 | 1.61E-04 |  |
| 7 | STX1A | 57 | 72751475 | 72771924 | 5.E-04 | rs12531606 | 1.61E-04 | integration of energy metabolism (Reactome), regulation of insulin secretion (Reactome), |
| 9 | ABO | 187 | 135120383 | 135140451 | 5.E-04 | rs495828 | 3.80E-04 |  |
| 10 | C10orf97 | 132 | 15860180 | 15942525 | 8.E-04 | rs7082146 | 6.72E-04 |  |
| 11 | UBE2L6 | 73 | 57075704 | 57092029 | 8.E-04 | rs2509897 | 6.05E-06 |  |
| 11 | SERPING1 | 78 | 57121602 | 57138902 | 1.E-04 | rs2509897 | 6.05E-06 | serine-type endopeptidase inhibitor activity (GO term), complement activation, classical pathway (GO term), intrinsic pathway (BioCarta), formation of fibrin clot clotting cascade (Reactome), formation of platelet plug (Reactome), intrinsic pathway (Reactome), platelet activation (Reactome), |
| 11 | YPEL4 | 61 | 57169135 | 57173993 | 9.E-04 | rs2509897 | 6.05E-06 |  |

**Table S11 Gene set enrichment analysis.** Details of gene sets with an empirical P value of < 5.E-3 using a 75% or 95% centile leading edge fraction cut-off are shown. No gene sets reached significance after correction for multiple testing (P <1.55E-5). Genes with a gene-based P value < 0.05 are shown for their corresponding gene set. N genes, number of genes assigned to pathway; FDR, false discovery rate; Exp gene, expected number of genes above cut-off; Obs gene, observed number of genes above cut-off.

| Database | Gene set | N genes | P value (95%) | FDR (95%) | Exp gene > 95% | Obs gene > 95% | P value (75%) | FDR (75%) | Exp gene > 75% | Obs gene > 75% | Genes |
| --- | --- | --- | --- | --- | --- | --- | --- | --- | --- | --- | --- |
| GO term | hormone-mediated signaling pathway | 52 | 0.0013 | 1.000 | 1 | 4 | 1.0E-04 | 0.334 | 3 | 10 | ADCY1, ADCY9, PRKAR1B, GNG13, GNG12 |
| GO term | lipid transport | 62 | 0.0027 | 1.000 | 1 | 4 | 0.1983 | 1.000 | 3 | 5 | CHKA, HDLBP, LDLR, ABCA12, OSBPL7 |
| GO term | protein tetramerization | 10 | 0.0048 | 1.000 | 0 | 2 | 0.0875 | 1.000 | 1 | 2 | CAT, SHMT1, AASS |
| Reactome | glucagon signaling in metabolic regulation | 34 | 0.0051 | 0.407 | 0 | 3 | 0.0002 | 0.039 | 2 | 8 | ADCY9, ADCY1, GNG12, GNG13, PRKAR1B |
| Reactome | gs alpha mediated events in glucagon signalling | 27 | 0.0290 | 0.545 | 0 | 2 | 0.0004 | 0.072 | 1 | 7 | ADCY9, ADCY1, GNG12, GNG13 |
| GO term | neuroblast proliferation | 14 | 0.1389 | 1.000 | 0 | 1 | 0.0035 | 0.719 | 1 | 4 |  |
| Reactome | thromboxane signalling through tp receptor | 21 | 0.1824 | 1.000 | 0 | 1 | 0.0039 | 0.106 | 1 | 5 | GNG13, GNG12 |
| Reactome | adp signalling through p2y purinoceptor 1 | 23 | 0.2045 | 1.000 | 0 | 1 | 0.0039 | 0.104 | 1 | 5 | GNG12, SRC, PLA2G4A, GNG13 |
| Reactome | thrombin signalling through proteinase activated receptors | 24 | 0.2169 | 1.000 | 0 | 1 | 0.0007 | 0.065 | 1 | 6 | GNG12, GNG13 |
| Reactome | signal amplification | 29 | 0.2504 | 1.000 | 0 | 1 | 0.0019 | 0.093 | 1 | 6 | GNAI2, GNG12, SRC, PLA2G4A, GNG13 |
| Reactome | glucagon type ligand receptors | 32 | 0.2722 | 0.969 | 0 | 1 | 0.0008 | 0.066 | 2 | 7 | GNG12, VIPR2, VIPR1, GNG13 |
| Reactome | platelet activation triggers | 56 | 0.4255 | 1.000 | 1 | 1 | 0.0016 | 0.097 | 3 | 9 | PLA2G4A, PRKCZ, AKT3, GNG13, GNAI2, GNG12, SRC, PIK3CB |
| GO term | anchored to plasma membrane | 16 | 1.0000 | 1.000 | 0 | 0 | 0.0011 | 0.512 | 1 | 5 | GAS1, RHBG, RTN4RL2 |

Further association analysis

Two SNPs in the TLR4 gene (rs2770150, rs4986790) have been shown to be associated with antibody response to pertussis toxin after vaccination([4](#_ENREF_4), [5](#_ENREF_5)). We examined the level of association with reported history of whooping cough in this sample at these SNPs.

**Table S12 Assessment of loci previously associated with whooping cough endophenotypes.** Odds ratios (OR) and 95% CIs (confidence intervals) are shown for a one unit increase in the number of effect alleles at each SNP within each cohort. The respective effect alleles are both G and their allele frequencies were 95% and 71% in the ALSPAC cohort and 94% and 71% in the 1958 Birth Cohort. Imputation quality was > 0.99 for each of these SNPs in each of the two cohorts.

|  |  | **ALSPAC** | | **1958 birth cohort** | |
| --- | --- | --- | --- | --- | --- |
|  | **SNP** | **OR (95% CIs)** | **P value** | **OR (95% CIs)** | **P value** |
| TLR-4 | rs4986790 | 1.11 (0.88, 1.39) | 0.390 | 0.97 (0.76, 1.22) | 0.772 |
|  | rs2770150 | 0.98 (0.88, 1.10) | 0.769 | 1.1 (0.97, 1.23) | 0.136 |

Supplemental references

1 Lee, S.H., Wray, N.R., Goddard, M.E. and Visscher, P.M. (2011) Estimating missing heritability for disease from genome-wide association studies. *Am. J. Hum. Genet.*, **88**, 294-305.

2 Wallenstein, S., Hodge, S.E. and Weston, A. (1998) Logistic regression model for analyzing extended haplotype data. *Genet. Epidemiol.*, **15**, 173-181.

3 Fisher, R.A. (1936) *Statistical methods for research workers*. Oliver & Boyd, Edinburgh;London.

4 Grondahl-Yli-Hannuksela, K., Vuononvirta, J., Barkoff, A.M., Viander, M., Van Der Meeren, O., Mertsola, J. and He, Q. (2012) Gene polymorphism in toll-like receptor 4: effect on antibody production and persistence after acellular pertussis vaccination during adolescence. *J. Infect. Dis.*, **205**, 1214-1219.

5 Banus, S., Bottema, R.W., Siezen, C.L., Vandebriel, R.J., Reimerink, J., Mommers, M., Koppelman, G.H., Hoebee, B., Thijs, C., Postma, D.S. *et al.* (2007) Toll-like receptor 4 polymorphism associated with the response to whole-cell pertussis vaccination in children from the KOALA study. *Clin. Vaccine Immunol.*, **14**, 1377-1380.
